# Impact of COVID-19 Social Distancing Restrictions on Training Habits, Injury, and Care Seeking Behavior in Youth Long-Distance Runners

**DOI:** 10.3389/fspor.2020.586141

**Published:** 2020-11-11

**Authors:** David M. Bazett-Jones, Micah C. Garcia, Jeffrey A. Taylor-Haas, Jason T. Long, Mitchell J. Rauh, Mark V. Paterno, Kevin R. Ford

**Affiliations:** ^1^Motion Analysis and Integrative Neurophysiology Laboratory, School of Exercise and Rehabilitation Sciences, University of Toledo, Toledo, OH, United States; ^2^Division of Occupational Therapy and Physical Therapy, Cincinnati Children's Hospital Medical Center, Cincinnati, OH, United States; ^3^Motion Analysis Lab, Division of Occupational and Physical Therapy, Cincinnati Children's Hospital Medical Center, Cincinnati, OH, United States; ^4^Doctor of Physical Therapy Program, San Diego State University, San Diego, CA, United States; ^5^Division of Sports Medicine, Cincinnati Children's Hospital Medical Center, Cincinnati, OH, United States; ^6^Deparment of Pediatrics, College of Medicine, University of Cincinnati, Cincinnati, OH, United States; ^7^Department of Physical Therapy, Congdon School of Health Sciences, High Point University, High Point, NC, United States

**Keywords:** adolescent, track and field, cross-country, overuse, health care provider, athletic trainer

## Abstract

**Purpose:** The COVID-19 pandemic impacted the sporting and exercise activities of millions of youth. Running is an activity that could be maintained while social distancing restrictions were implemented during the pandemic. If running-related injuries do occur, these restrictions may also influence the access to care or care seeking behavior of this population. Therefore, the purpose of this study was to determine if the social distancing restrictions during the 2020 COVID-19 pandemic influenced training habits, injury, and care seeking behavior in youth long-distance runners.

**Methods:** A customized, open online questionnaire was provided to runners 9–19 years of age who participated in long-distance running activities including team/club cross-country, track and field (distances ≥800 m), road races, or recreational running. Participants responded to questions about demographics, running habits, RRIs, and health care provider visits 6-months before as well as during social distancing restrictions due to COVID-19. Wilcoxon signed rank tests compared differences for ratio data and Chi-square tests were used to compare proportions before and during COVID-19 social distancing restrictions. Statistical significance was set at *p* ≤ 0.05.

**Results:** A total of 287 youth long-distance runners (male = 124, female = 162, unspecified = 1; age = 15.3 ± 1.7 years; running experience = 5.0 ± 2.3 years) participated. Compared to their pre-COVID-19 responses, youth long-distance runners reported lower distances run per week (*p* < 0.001), fewer runs per week (*p* < 0.001), fewer hard runs per week (*p* < 0.001), fewer number of injuries (*p* < 0.001), and fewer injuries per 1,000 km (*p* = 0.002) during the COVID-19 social distancing restrictions. A lower proportion of participants reported in-person health care provider visits (*p* < 0.001) and a lower proportion of visits were made to an athletic trainer during COVID-19 social distancing restrictions compared to prior to COVID-19 (*p* < 0.001).

**Conclusion:** The COVID-19 pandemic resulted in significant decreases in both training and injuries which were different compared to previous reports in an adult population. Many of the runners who sustained an injury during COVID-19 social distancing restrictions did not seek care, with the most prominent reduction in visits to an athletic trainer. This could impact future injury and chronic pain.

## Introduction

On March 11th, 2020, the World Health Organization (WHO) declared Coronavirus Disease 2019 (COVID-19) a pandemic (World Health Organization, 2020). In response to the pandemic, and in an effort to minimize the rate of new infections, policies of social distancing and shelter in place became commonplace in many national, regional, and local locations. Schools at all levels canceled in-person classes, as well as extracurricular social and athletic activities. Non-essential businesses that offered a high risk of transmission like gyms and exercise facilities also closed. Millions of youth who are typically physically active through interscholastic or club sports were suddenly required to stay at home. Running is a mode of exercise that may be performed outside of facilities and with appropriate social distancing. Thus, while youth runners could have potentially maintained their pre-COVID-19 running habits, it is unknown if they maintained their running activities during the COVID-19 restrictions.

Partly due to its ease and accessibility, running is one of the most popular forms of physical activity in youth (Fakhouri et al., 2014). Track and field and cross-country constitute the first and sixth most popular high school sports (respectively) in the United States, totaling over 1.5 million combined participants (National Federation of State High School Associations, 2019). They also have the greatest number of participants in middle school athletics (Beachy and Rauh, 2014). While running provides benefits to both physical and mental health (Baghurst et al., 2015), high participation rates in running are concomitant with high rates of running-related injury (RRI). More than one out of every three high-school runners sustains a RRI during the course of a cross-country season (Rauh et al., 2006) and 59–68% of runners report a history of RRI (Tenforde et al., 2011). Given the cancelation of practices and competition seasons during the COVID-19 pandemic, it seems likely that injury risk would be impacted by reduced training volume. While the risk of RRI in adults appears to have been heightened during the COVID-19 social distancing restrictions (DeJong et al., 2020), to our knowledge, there are no reports on its impact in youth long-distance runners.

The most common type of injury in youth long-distance runners are overuse injuries (Tenforde et al., 2011). These injuries are often linked to excessive training volumes, higher training intensities, and other training-related errors (Tenforde et al., 2011). If not appropriately managed, many of these injuries have the potential to increase in chronicity. Therefore, access to healthcare providers and care seeking behavior are important considerations in youth long-distance runners. The closure of schools limited students' access to school-based athletic trainers, and due to the COVID-19 pandemic, many health care provider offices closed or transitioned to a telemedicine-based model (e.g., phone or video calls). While telehealth in sports medicine is feasible and patients have reported a positive experience during COVID-19 (Tenforde et al., 2020), it is unknown if youth long-distance runners have utilized telehealth to seek care for RRIs sustained during social distancing restrictions.

The purpose of this study was to determine if the social distancing restrictions during the 2020 COVID-19 pandemic influenced training habits, injury, and care seeking behavior in youth long-distance runners. We hypothesized that (1) self-reported weekly running distance, frequency, and intensity would decrease during restrictions, (2) social distancing restrictions would result in fewer injuries, and (3) injured long-distance runners would report more telehealth appointments and fewer in-person appointments with health care providers; athletic trainers would see the greatest reduction in health care encounters.

## Materials and Methods

### Participants

Youth long-distance runners were recruited via emails sent to middle- and high-school athletic directors or coaches across the United States and previous participants in youth long-distance running studies from two institutions. Recruitment materials were initially sent May 2020 through June 2020 with a secondary invitation sent between 10 and 14 days after the initial invitation. The survey link was also shared on various social media platforms and others were encouraged to share the survey. Participants were eligible for study inclusion if they were 9–19 years of age and participated in long-distance running activities including team/club cross-country, track and field (distances ≥800 m), road races, or recreational running. Participants were excluded if their primary sport was not cross-country or track and field and they did not participate in long-distance running activities and/or if they resided outside of the United States. Study procedures were approved by the Institutional Review Boards at the University of Toledo and Cincinnati Children's Hospital Medical Center. Prior to study enrollment, consent was obtained for participants 18 or 19 years of age while parental permission and child assent were obtained for those 9–17 years-old. The participants received no incentives for participating.

### Questionnaire

A customized, open online questionnaire was provided to the participants through a password-protected Qualtrics (SAP SE, Germany) or Research Electronic Data Capture (REDCap, Vanderbilt University, TN) instrument. The Checklist for Reporting Results of Internet E-Surveys (CHERRIES) was used to ensure study quality (Eysenbach, 2004). Questions were exactly the same for both instruments and institutions. The questionnaire required ~10 min to complete and consisted of 83 items, split up among a minimum of 13 screens, related to demographics (age, sex, state, years of running experience, recreational/competitive runner), running habits, RRIs, and health care provider visits 6-months before as well as during social distancing restrictions due to COVID-19 (DeJong et al., 2020). Questions were presented in a consistent order with adaptive questioning dependent on participant responses. Participants were given as much time as necessary to complete the survey. Participants had the opportunity to review and change their responses prior to submitting them. Once the submission was made, no changes to their responses were allowed.

Running habits questions included self-reported average weekly running distance (miles), average number of runs per week, and average number of higher intensity runs per week (defined as heavy breathing, and difficulty talking while running) (Persinger et al., 2004). RRIs were assessed by asking participants if they had incurred or were presently experiencing a RRI (yes/no) during this time period, and if so, report the number of RRIs sustained. Injured runners were asked to complete a matrix with the body location (toe, foot, ankle, lower leg, knee, thigh, hip/pelvis, groin, abdomen, back, other) and injury type. Injury types included muscle injury (i.e., “pulled” muscle, muscle strain), tendon injury (i.e., tendonitis, tendon tear), bone injury (i.e., stress fracture, fracture), and ligament injury (i.e., sprain).

Participants who indicated that they had sustained an injury were presented with follow-up questions regarding their health care provider visit(s). Injured runners were asked about the type of health care visit they sought (in-person, telehealth, or no visit). Telehealth visits included remote video or audio calls. Respondents were also asked about the provider specialty that the participant visited for the injury (pediatrician, podiatrist, chiropractor, physical therapist, athletic trainer, sports medicine primary care physician, orthopedic surgeon, none, other).

Incomplete responses were excluded and completed surveys were used for analysis. The IP address was recorded for each response. If duplicate IP addresses were identified, the responses were checked for originality and responses from the same IP address were only used if the responses were clearly different. Weekly running distance was reported in miles and then converted to kilometers (km). The number of injuries for each person were normalized to 1,000 km to reduce the impact that running volume could have on the injury rate.

### Statistical Analyses

Data analysis was conducted using SPSS statistical software (version 26, IBM Inc., Armonk, NY). Data were found to be non-normally distributed (Kolmogorov-Smirnov, *p* ≤ 0.05). Wilcoxon signed rank tests compared differences before and during COVID-19 social distancing restrictions for distance run per week, number of runs per week, number of hard runs per week, number of injuries and number of injuries per 1,000 km. For participants that reported ≥1 RRI before and/or during COVID-19 social distancing restrictions, chi-square tests compared differences in proportions of health care providers and visit types before and during COVID-19 social distancing restrictions. Statistical significance was set at *p* ≤ 0.05.

## Results

**Figure 1:**
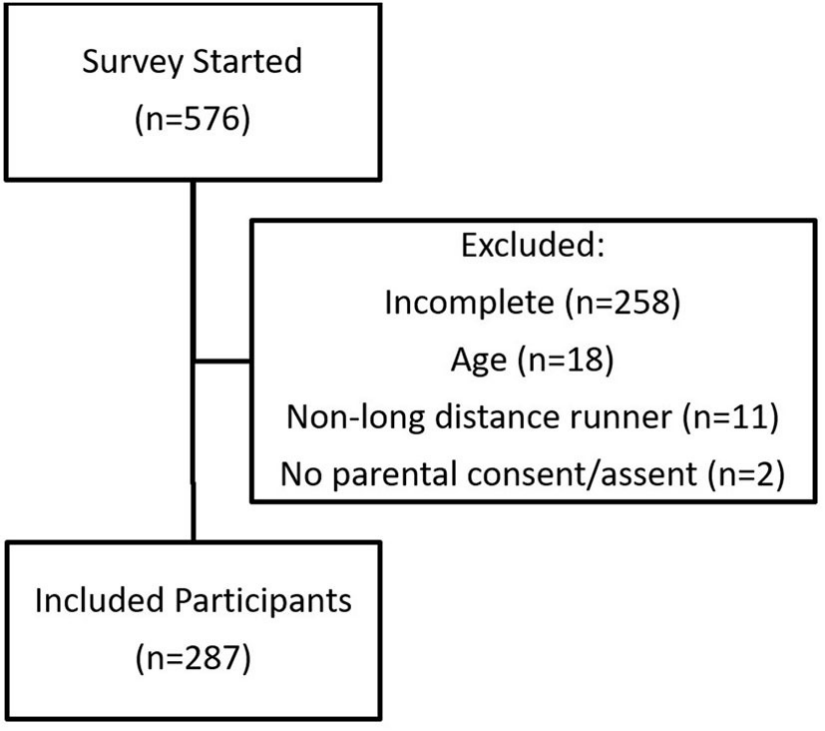
Flow chart of study participants.

Of the 576 survey attempts, 287 (male = 124, female = 162, unspecified = 1; age = 15.3 ± 1.7 years; running experience = 5.0 ± 2.3 years) met inclusion criteria and were fully completed (completion rate = 49.8%; Figure 1). The majority of the excluded responses were due to being outside the age range of the current study. In total, 235 (81.9%) and 52 (18.1%) participants reported they were competitive and recreational runners, respectively. Participants resided in 20 different states with a majority coming from the Midwest United States (Table 1). Compared to their pre-COVID-19 responses, youth long-distance runners reported lower distances run per week (*p* < 0.001), fewer runs per week (*p* < 0.001), and fewer hard runs per week (*p* < 0.001) during the COVID-19 social distancing restrictions (Table 2).

**Table 1:** Number and percentage of completed responses by state.

**State**	***N***	**Percent of responses**
OH	82	28.6%
IL	43	15.0%
WI	41	14.3%
ID	18	6.3%
WA	17	5.9%
KY	17	5.9%
NC	16	5.6%
Other^*^	53	<5% each

* *AR, AZ, CA, CT, KS, MN, NE, NV, NY, SC, TN, UT, WV*.

**Table 2:** Running habits and injury incidence before and during COVID-19 social distancing restrictions.

**Variable**	**Median [25th, 75th percentile]**		**Mean [Standard deviation]**		P^*^
	**Pre-COVID-19**	**During-COVID-19**	**Pre-COVID-19**	**During-COVID-19**	
Weekly distance run [km]	37.0 [24.1, 56.3]	31.4 [12.0, 48.3]	38.6 [22.0]	33.2 [24.2]	<0.001
Runs per week [*n*]	5.0 [5.0, 6.0]	5.0 [3.0, 6.0]	5.0 [1.4]	4.3 [2.0]	<0.001
Hard runs per week [*n*]	3.0 [2.0, 3.0]	2.0 [1.0, 3.0]	2.6 [1.2]	1.9 [1.5]	<0.001
Injuries [*n*]	0.0 [0.0, 0.0]	0.0 [0.0, 0.0]	0.3 [0.6]	0.2 [0.4]	<0.001
Injuries [*n*/1,000 km]	0.0 [0.0, 0.0]	0.0 [0.0, 0.0]	11.4 [36.5]	6.5 [26.2]	0.002

* *Wilcoxon signed rank statistical significance*.

Youth long-distance runners also reported fewer number of injuries (*p* < 0.001) and fewer injuries per 1,000 km (*p* = 0.002) than pre-COVID-19 social distancing restrictions (Table 2). Prior to COVID-19 social distancing restrictions, 61 (21.3%) participants reported 120 RRIs while 41 (14.3%) participants reported 82 RRIs during COVID-19. Pre-COVID-19, the most common injury locations were the lower leg (19.2%), knee (18.3%), and hip/pelvis (15.8%); whereas during COVID-19 social distancing restrictions, the most common injury locations were the knee (18.3%), hip/pelvis (18.3%), and lower leg (17.1%) (Table 3).

**Table 3:** Number of self-reported injuries before and during COVID-19 social distancing restrictions by location and injury type.

		**Muscle injury**		**Tendon injury**		**Bone injury**		**Ligament injury**		**Total injuries**	
		**Before**	**During**	**Before**	**During**	**Before**	**During**	**Before**	**During**	**Before**	**During**
Toe		3	0	1	1	2	0	3	2	9 [7.5%]	3 [3.7%]
Foot		2	1	4	5	3	2	3	1	12 [10.0%]	9 [11.0%]
Ankle		4	2	4	4	1	2	4	4	13 [10.8%]	12 [14.6%]
Lower leg		7	5	5	4	11	5	0	0	23 [19.2%]	14 [17.1%]
Knee		6	5	10	8	1	0	5	2	22 [18.3%]	15 [18.3%]
Thigh		10	6	1	0	0	0	0	0	11 [9.2%]	6 [7.3%]
Hip/pelvis		9	7	5	4	4	1	1	3	19 [15.8%]	15 [18.3%]
Groin		4	2	0	0	0	0	0	0	4 [3.3%]	2 [2.4%]
Abdomen		1	1	0	0	1	0	0	0	2 [1.7%]	1 [1.2%]
Back		4	4	0	1	0	0	0	0	4 [3.3%]	5 [6.1%]
Other		0	0	0	0	1	0	0	0	1 [0.8%]	0 [0.0%]
Total	*n*	50	33	30	27	24	10	16	12	120 [100.0%]	82 [100.0%]
	%	41.7	40.2	25.0	32.9	20.0	12.2	13.3	14.6		

COVID-19 social distancing restrictions impacted the proportion of youth long-distance runners seeking care for their injuries compared to pre-COVID-19 behaviors. A lower proportion of participants reported in-person health care provider visits (*p* < 0.001) and a higher proportion of not visiting a health care provider (*p* < 0.001), while the proportion of telehealth visits did not change (*p* = 0.81, Table 4). Although the proportion of visits to most healthcare providers did not change (Table 5), a significantly lower proportion (22% of visits; *p* < 0.001) of visits were made to an athletic trainer during COVID-19 social distancing restrictions compared to prior to COVID-19 (56% of visits).

**Table 4:** Proportion of health care provider visit types before and during COVID-19 social distancing restrictions in injured runners.

**Variable**	**Pre-COVID-19**	**During-COVID-19**	P^*^
	***n* = 63**	***n* = 42**	
**In-person visit**, ***n*** **[%]**	**51 [80.9]**	**17 [40.5]**	**<0.001**
Telehealth visit, *n* [%]	2 [3.2]	1 [2.4]	0.81
**No visit**, ***n*** **[%]**	**10 [15.9]**	**24 [57.1]**	**<0.001**

* *Chi-square statistical significance. **Bold**=p ≤ 0.05*.

**Table 5:** Proportion of health care provider visits before and during COVID-19 social distancing restrictions in injured runners.

**Variable**	**Pre-COVID-19**	**During-COVID-19**	P^*^
Athletic trainer, *n* [%]	**40 [59.7]**	**9 [22.0]**	**<0.001**
Chiropractor, *n* [%]	10 [14.9]	6 [14.6]	0.97
Orthopedic surgeon, *n* [%]	9 [13.4]	4 [9.8]	0.57
Pediatrician, *n* [%]	5 [7.5]	4 [9.8]	0.68
Physical therapist, *n* [%]	21 [31.3]	14 [34.1]	0.76
Sports medicine physician, *n* [%]	28 [41.8]	17 [41.5]	0.97
Podiatrist, *n* [%]	3 [4.5]	2 [4.9]	0.92

* *Chi-square statistical significance. **Bold**=p ≤ 0.05*.

## Discussion

The purpose of this study was to determine if the social distancing restrictions during the 2020 COVID-19 pandemic influenced training habits, injury, and care seeking behavior in youth long-distance runners. We found that COVID-19 social distancing restrictions significantly impacted youth long-distance runners' training habits, injuries, and care seeking behaviors. These changes, which supported our hypotheses, could have implications for the health and well-being of this population in the short and long-term, which requires further investigation.

Youth long-distance runners decreased their weekly running distance, number of weekly runs, and number of weekly hard runs during COVID-19 social distancing restrictions. Due to the COVID-19 pandemic, the track and field seasons in the spring of 2020 were canceled. This running population is likely strongly influenced by intrinsic psychological and social factors such as the desire to compete and run with their teammates (Yan and McCullagh, 2004), as well as extrinsic factors such as motivation provided by their coaches and parents (Goose and Winter, 2012). The absence of these motivating factors may have contributed to the reduced training volume and intensity. The responses by youth long-distance runners in our study are different than the responses of adult runners during the COVID-19 pandemic, which observed an increase in running volume (DeJong et al., 2020). This increase in running among adults was largely thought to be influenced by a desire to maintain health instead of competition (DeJong et al., 2020). Youth long-distance runners may have a strong desire to return to pre-COVID-19 volume and intensity levels with the goal of returning to competition in the fall of 2020; however, sudden changes in these variables could have led to overuse injury (Nielsen et al., 2012, 2013, 2014). Coaches and health care providers who interact with this population should be aware that motivators in this population may make youth long-distance runners vulnerable to overuse injury as they return to a competitive season.

The number of reported injuries significantly decreased in youth long-distance runners during the COVID-19 pandemic. However, even when normalized to running distance, self-reported injuries were significantly decreased. This injury reduction may have been influenced by the decrease in training volume and intensity since injury has been linked to these variables in youth long-distance runners (Tenforde et al., 2011). Contrary to our results, adult runners experienced an increase in running injury risk during COVID-19 (DeJong et al., 2020). While novice adult runners have demonstrated excellent specificity and sensitivity for self-reporting injury location compared to physician-reports (Smits et al., 2019), it has not been reported whether youth runners are also able to accurately self-report RRIs. Youth are less likely to report an injury that is chronic pain/overuse-related. Thus, they may have been more likely to report injuries that were more traumatic or acute in nature (Rathleff et al., 2013), thereby decreasing the overall number of injuries reported. Due to the cross-sectional design of this study, we were unable to further elucidate if this decrease in injury was actual or spurious due to other factors.

Health care provider visits and care seeking behavior changed during the COVID-19 pandemic, with a decrease in visits to health care providers. Prior to the COVID-19 social distancing restrictions, youth long-distance runners reported that most (83.6%) of their visits were in-person and only a small amount of injuries were not brought to the attention of a health care provider (16.4%). Youth runners significantly reduced their visits to health care providers during the COVID-19 restrictions (41.5%) and were 3.6 times more likely to not visit a health care provider (58.5%) for their injury. Young runners may rely on their parents for transportation or assistance with provider interactions, creating an additional burden to care seeking behaviors. Telehealth appointments are a feasible alternative to in-person appointments (Tenforde et al., 2020) and could reduce these barriers to youth. However, very few of the youth runners in this study participated in telehealth appointments before (3.3%) or during (2.4%) the COVID-19 pandemic. Many of these injuries, if not treated, are thought to lead to long-term, chronic conditions (Thomas et al., 2010) and this could be further exacerbated by the COVID-19 pandemic (Clauw et al., 2020). These data also suggest the need to develop plans to maintain access to medical services in the event of similar future sport activity (running) stoppages and/or restrictions. An interdisciplinary team, including policy makers, school administrators, and/or national associations, should consider developing these plans in advance to ensure access to care. Further, coaches and health care professionals may want to devise safe return-to-running protocols that may minimize the risk of running-related injuries.

The reduction in visits were primarily evident in reported visits to athletic trainers. Injured youth long-distance runners utilized an athletic trainer significantly less for their health care needs during the COVID-19 pandemic. Youth runners sought care from athletic trainers 2.7 times less during this period, though care seeking behavior from other providers did not change. Approximately 76% of high schools employ an athletic trainer (Huggins et al., 2019); however, as sports seasons were canceled early, access to these providers were potentially limited to these injured runners. Increased utilization of telemedicine by athletic trainers and other health care providers could improve access to care for this population during restrictions or in rural/remote environments (McLeod et al., 2013). Further research is needed to determine the specific reasons that youth long-distance runners seek care, the mediums by which they seek care, and which provider(s) they choose to seek care from.

The primary limitation of the current study is the cross-sectional design of an online survey where recall bias could influence the reporting of training habits and injuries. We attempted to minimize the impact of this bias by limiting the recall period to 6 months prior to COVID-19. Social distancing restrictions also differed in extent and timeline based on state government decisions, leading to likely variability in the stay-at-home orders affecting each participant. This study is also limited in that we only assessed the runners' responses at one time point. Future research should prospectively investigate training habits and injuries in a larger group of youth runners in the season following the COVID-19 restrictions to provide evidence of a long-lasting impact in this running population. Finally, even though we attempted to get a broad sense of the COVID-19 impact on the runners, the responses were weighted more heavily among a few US states which could also have introduced a geographic bias. Future research should attempt to capture a more balanced sampling of responses of youth runners in different regions of the US to better examine any geographic influence. In regard to COVID-19, future research should follow individuals over time to determine the influence of social distancing, reduced running, and reduced care-seeking behavior has on long-term running health and habits.

Within the context of these limitations, this study provides novel information about the effects that a large-scale cancellation of a sports season and social distancing restrictions may have on youth long-distance runners. Following social distancing requirements from the COVID-19 pandemic, youth long-distance runners reported significant decreases in both training and injuries, which were different compared to previous reports in an adult population (DeJong et al., 2020). Many of the runners who sustained an injury during COVID-19 social distancing restrictions did not report seeking care, with the most prominent reduction in visits to an athletic trainer. This could impact future injury risk and chronic pain if appropriate care is not provided for this young population.

## Data Availability Statement

The raw data supporting the conclusions of this article will be made available by the authors, without undue reservation.

## Ethics Statement

The studies involving human participants were reviewed and approved by University of Toledo Institutional Review Board and Cincinnati Children's Hospital Medical Center Institutional Review Board. Written informed consent to participate in this study was provided by the participants' legal guardian/next of kin.

## Author Contributions

DB-J, MG, JT-H, JL, MR, MP, and KF designed the study, contributed to data collection and analysis, and critically reviewed the manuscript before submission. All authors contributed to the article and approved the submitted version.

## Conflict of Interest

The authors declare that the research was conducted in the absence of any commercial or financial relationships that could be construed as a potential conflict of interest.
